# Reducing microbial contamination on historical leather artifacts at the Auschwitz-Birkenau State Museum using ethanol in the form of mist

**DOI:** 10.3389/fmicb.2025.1576114

**Published:** 2025-04-10

**Authors:** Krzysztof Kraśnicki, Natalia Pydyn, Aleksandra Papis, Krystyna Guzińska, Dorota Kaźmierczak, Urszula Maciołek, Anna Wawrzyk

**Affiliations:** ^1^Auschwitz-Birkenau State Museum, Oświęcim, Poland; ^2^Lukasiewicz Research Network-Lodz Institute of Technology, Lodz, Poland; ^3^Analytical Laboratory, Institute of Chemical Sciences, Faculty of Chemistry, Maria Curie-Skłodowska University, Lublin, Poland; ^4^Department of Basic Biomedical Science, Faculty of Pharmaceutical Sciences in Sosnowiec, Medical University of Silesia, Sosnowiec, Poland

**Keywords:** ethanol, biodeterioration, microorganisms, cultural heritage, disinfection

## Abstract

**Purpose:**

The aim of the study was to evaluate the biocidal efficacy and determine the influence of 90% ethyl alcohol applied in the form of a mist on the surface of model and historical leather.

**Materials and methods:**

The main object of the study were historical leather shoes from the collections of the Auschwitz-Birkenau State Museum (A-BSM) in Oświęcim (Poland). Microorganisms found before on historical leather objects in A-BSM were inoculated onto samples of model and historical leather. Ethanol mist was applied with an airbrush with optimized parameters for 15 s at a concentration of 90%. To increase the biocidal effectiveness, the samples were sealed in a tight package and stored for 22 hours. The effect of disinfection was assessed using culture-dependent methods. Changes on the surfaces was assessed using SEM, FTIR, and XPS techniques.

**Results:**

On surfaces inoculated with microorganisms in the following quantities: 10^5^ − 10^8^ CFU bacteria and 10^5^ CFU fungi, a reduction of 99.51% to 99.99% was observed. Ethanol disinfection had no negative effect on the surface morphology and collagen structure.

**Conclusions:**

Disinfection with ethanol applied in the form of mist can be effectively used to eliminate microbiological contamination of historical objects made of leather in A-BSM.

## 1 Introduction

The German Nazi concentration and extermination camp Auschwitz-Birkenau was established in 1940. It was an important part of the drama of World War II and the result of the genocidal policy of Nazi Germany. The huge number of victims, the vast area from which people were brought, the industrial methods of killing people in gas chambers made A-BSM the most recognizable symbol of the Holocaust and the site of Genocide in the world (Ambrosewicz-Jacobs et al., 2008).

**Figure 1:**
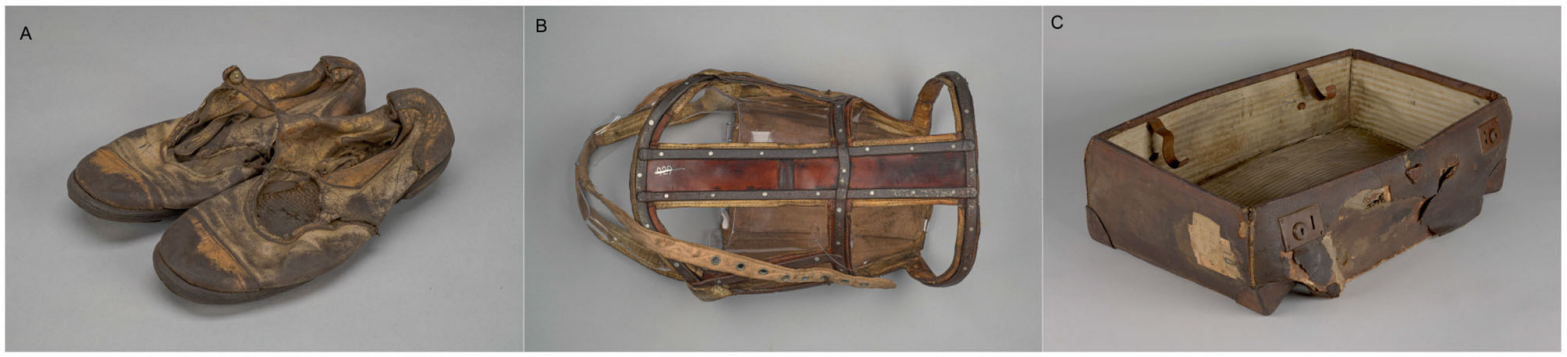
Objects made of leather from the collections of the A-BSM in Oświęcim **(A)** shoes **(B)** orthopedic prosthesis **(C)** suitcase. Photo: Sebastian Mrozek.

In order to preserve the memory of the victims of the Nazi regime, the A-BSM was established in the former camp area. The institution's activities focus on securing the remains of the former camp, including personal items found after the camp was liberated, which are often the only evidence of the presence of the people who were imprisoned there or killed in gas chambers. The stolen property consists largely of items containing leather elements. These include shoes, suitcases, clothing and equipment, as well as orthopedic supplies. Leather shoes are one of the largest groups of objects in the Auschwitz Museum Collections (Figure 1).

The Museum's activities focus on ensuring proper storage conditions and conducting necessary conservation work on the remains of the camp and its facilities. All facilities and their elements require protection against deterioration processes in a way that causes the least possible damage to their surfaces.

The metabolic activity of microorganisms is the one of the factors causing the destruction of museum collections. Contamination of historic objects is not only the result of microflora floating in the air, although this microflora, including bacteria and fungi, may affect the state of preservation of such objects. There are also other factors that contribute to the degradation of historic objects, including primary contamination. Highly porous materials are more susceptible to microbial colonization because of their capacity to absorb more water and retain it in for longer periods of time. Surface roughness affects the trapping of moisture and concentrates it in micro-fissures where growth is usually more abundant. It also enables the accumulation of particles - soot, organic and inorganic debris, pollens, spores, and salts (Jacob et al., 2018). Microflora transferred by air movement onto historical objects gradually inhabits the microstructural discontinuities of the surface and, through the emission of extracellular enzymatic substances, causes their hydrolysis to simple organic compounds. The biodeterioration of objects is initiated by the naturally occurring enzymatic activity of microorganisms due to the secretion of enzymes from groups such as cellulases, amylases, and proteases. As a result of microbiological decomposition, discolouration, and biological deposits may appear on historical objects, leading to structural weakening and disruption of the continuity of the surface (Szostak-Kotowa, 2004).

As a result of the conducted studies on the identification of microorganisms occurring on historical objects in A-BSM several species of bacteria were identified, including: *Bacillus atrophaeus, Bacillus cereus, Bacillus licheniformis, Bacillus megaterium, Bacillus simplex, Bacillus subtilis, Enterobacter* sp., *Micrococcus luteus, Pantoea agglomerans, Psychrobacillus psychrodurans, Staphylococcus aureus, Staphylococcus epidermidis*, and the following species of fungi were identified: *Alternaria alternata, Aspergillus flavus, Aspergillus niger, Chaetomium globosum, Cladosporium cladosporioides, Epicoccum nigrum, Penicillium chrysogenum, Rhizopus nigricans, Stephanoascus cifferrii*, and *Trichoderma viride* (Wawrzyk et al., 2018).

Microorganisms, due to complex adaptation mechanisms to changing environmental conditions, are characterized by a wide range of tolerance to changes in pH, temperature and humidity, and a small amount of organic substances is enough for them to survive and develop. Materials of animal or plant origin are particularly susceptible to decomposition, i.e., those made of leather, wood, paper, silk, or wool. The mechanism of biological decomposition of organic materials is based on metabolic activity due to the action of ligninocellulolytic enzymes in the case of wood and paper, cellulases, keratinases, and esterases in the case of leather materials and clothing (Mazzoli et al., 2018).

Therefore, a very important stage preceding the conservation of historical objects is their examination and if there is such need—decontamination. Currently, various disinfection methods are used in museums. These include: washing with alcohol, spraying with quaternary ammonium compounds or fogging with ethylene oxide. Many of the biocidal substances used leave noticeable changes on the surface of the disinfected object, which is not permissible in the case of historical materials (Sequeira et al., 2012; Gutarowska et al., 2017). The effect of disinfection may be the opposite of the expected one and cause accelerated degradation processes (Ortiz et al., 2013).

Considering the conservation works, special attention should be paid to the selection of disinfection methods for historic objects that determine the safety of people performing renovation works. The disinfectant cannot pose a greater threat than the microorganisms it is used to eradicate. The disinfection commonly used in museums so far is often harmful to human health, e.g. ethylene oxide (ETO), highly effective and safe for most museum objects, but at the same time toxic and carcinogenic to humans (Wawrzyk et al., 2018).

Historical objects, depending on the material they are made of, are inhabited by microorganisms with varying sensitivity to disinfectants. Ethanol is a commonly known disinfectant in medicine. Liquid ethanol in concentrations ranging from 60% to 90% has biocidal properties against bacterial, fungal, and viral cells. Its action is based on the denaturation of microbial proteins, leading to the disruption of cell wall continuity and the spilling of cytoplasm into the extracellular environment (Gerba et al., 2024).

In medicine, ethanol is used in liquid form. In the case of museum objects, prolonged contact of aqueous solutions with historical surfaces is not recommended. Increased moisture level could be an initiator of accelerated degradation processes which would lead to irreversible changes in structure by dissolving compounds sensitive to contact with aqueous solutions. The molecular structure of ethanol, due to the presence of a hydroxyl group, makes it a substance with a high redox potential, capable to react with many organic substances, which is not desirable in the case of surfaces of historical objects (Dai et al., 2025). A concentration of 90% ethanol was introduced to accelerate evaporation from the surface and thus shorten the time of action on the surface in this study. Thanks to that, the humidity of the object would not increase, otherwise soaking of the surface could make them vulnerable to damage. The effectiveness of application of 90% ethanol in the mist form to disinfect textiles was demonstrated by us before (Wawrzyk et al., 2023).

Due to the biocidal effectiveness of liquid ethanol demonstrated in the use of disinfection of abiotic surfaces, the effect of ethanol was tested using a mist application.

The effectiveness of ethanol was studied using strains isolated from the Museum's objects and strains from the American Type Culture Collection (ATCC). The bacteria used were: *Pantoea agglomerans, Staphylococcus aureus*, and molds: *Alternaria alternata, Aspergillus niger, Penicillium chrysogenum*, and *Aspergillus flavus*. The species were selected based on their frequency of occurrence, potential harmful effects on human health, and adverse effects on objects. Fungi of the genera *Aspergillus, Alternaria*, and *Penicillium* can cause chronic inflammation in humans resulting from continuous exposure to mold spores, and can also contribute to severe respiratory infections (Hyde et al., 2018). *Staphylococcus aureus* may pose a threat to human health through direct contact with contaminated surfaces causing allergies and purulent leather infections (Tong et al., 2015). Microorganisms with the ability to enzymatically decompose cellulose and produce acids that contribute to the progress of bio-deterioration on objects (*A. alternata, A. flavus, A. niger*, and *P. chrysogenum*) were also selected for research (Di Carlo et al., 2016). Endotoxin produced by *Pantoea agglomerans* and other Gram-negative bacteria present in leather dust is considered to be the main cause of byssinosis, initiating acute and chronic inflammation of endothelial cells (Dutkiewicz et al., 2016).

Conservation and disinfection works carried out on historical objects cannot adversely affect their surface structure. Very precise, accurate analyses of the surfaces should be carried out before and after the use of the implemented disinfection techniques (Wawrzyk et al., 2020b). Comprehensive physicochemical analysis—FTIR (Fourier Transform Infrared Spectroscopy), SEM (Scanning Electron Microscopy), and XPS (X-ray photoelectron Spectroscopy) allowed for the assessment of possible changes in the collagen structure and determination of the condition of the layers covering the historical leather objects and thus to determine whether the disinfection using ethanol in the mist form negatively affected the surfaces of the tested leather surfaces.

## 2 Materials and methods

### 2.1 Microbiological examination of historical objects

For quantitative studies of microbial contamination of collagen objects in A-BSM, 16 children's shoes were used. Swabs were taken from the outer surfaces of footwear made of degraded collagen materials, vegetable-tanned grain leather that showed visible signs of biodegradation. Material for analysis was collected using sterile swabs from surfaces limited by a 25 cm^2^ template, and the swab was then placed in a transport tube. The number of microorganisms was determined using the culture method with serial dilutions. After transport to the laboratory, each collected sample was suspended in 1 ml of sterile 0.85% physiological saline solution and shaken thoroughly for 1 min. 100 μl of the sample was plated on plates with certified solid, ready-made microbiological media (BTL, Poland). Two different media were used: standard agar for determining the total number of bacteria Plate Counting Agar (PCA) and agar Malt Extract Agar (MEA) with malt extract for determination of the number of filamentous fungi and yeasts. The plates were incubated for 10 days at 26°C, and then the bacterial colonies and the microscopically identified mold fungi that grew on solid media were counted. The results were presented as the number of colony-forming units per 100 cm^2^ of the tested surface.

### 2.2 Preparation of inoculum and leather samples

**Figure 2:**
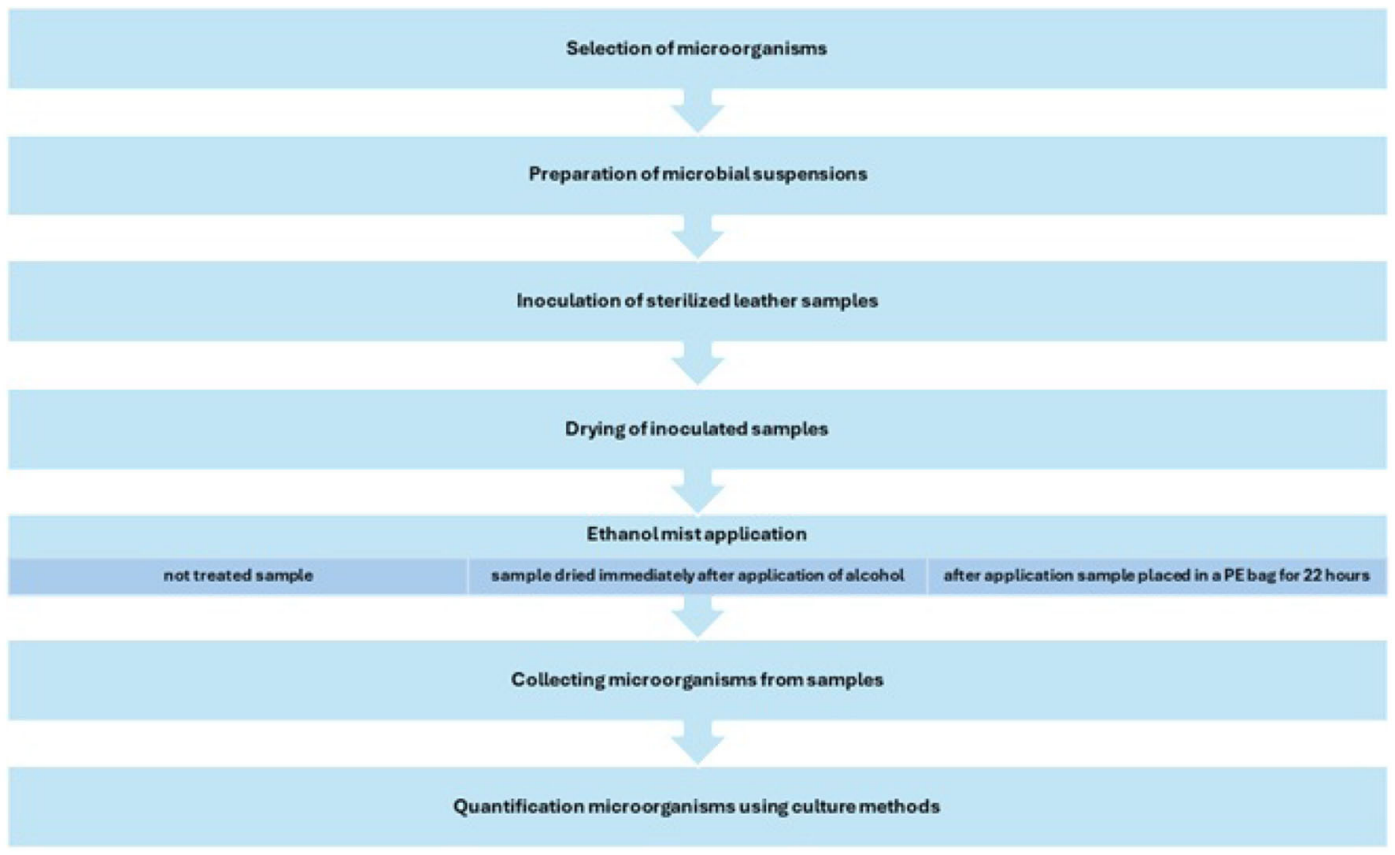
Diagram of biocidal effectiveness of ethanol in the form of mist research.

A diagram showing the biocidal effectiveness of ethanol in the form of mist research action plan is shown in the Figure 2.

Microorganisms from recognized culture collections (ATCC-American Type Culture Collection) and isolated from the surface of leather objects in the A-BSM collections were used to study the biocidal efficacy of ethanol. The microorganisms were isolated in previous studies, deposited in cryobanks of the A-BSM laboratory and, after multiplication, used in this work. The mold fungi selected for the study were inoculated on Sabouraud agar medium with 4% glucose (BTL, Poland). The plates were incubated at 25 ± 2°C for 5 days. Suspensions were prepared from the cultures in sterile water to obtain a density of approximately 10^5^ − 10^6^ CFU/sample. The suspension obtained was diluted tenfold each time and the number of CFU (Colony Forming Unit) was determined using the plate count method. The bacteria were inoculated on Plate Count Agar (BTL, Poland). The plates were incubated at 30 ± 2°C for 2 days. Suspensions were prepared from the cultures in sterile water, diluted in peptone water and determined using the plate count method to obtain a density of approximately 10^5^ − 10^8^ CFU/ml on the sample.

The concentrations of the microbial suspension were used as described in Table 1.

**Table 1:** Concentrations of microbial suspensions applied to model and historic leather samples.

**Strain**	**Concentration of microbial suspension [CFU/ml]**
*Alternaria alternata* (A-BSM cryobank)	5.5 × 10^5^
*Aspergillus flavus* (ATTC 9643)	1.85 × 10^6^
*Aspergillus niger* (A-BSM cryobank)	2.30 × 10^6^
*Pantoea agglomerans* (A-BSM cryobank)	4.59 × 10^8^
*Penicillium chrysogenum* (A-BSM cryobank)	1.88 × 10^6^
*Staphylococcus aureus* (ATTC 6538)	4.73 × 10^8^

Historical leather samples of leather shoe dated to the first half of the 20th century from A-BSM collection were used to optimize the process. Studies were also performed on modern vegetable-tanned pig grain leather, referred in this article as model leather. Historical and model leather samples measuring 40 mm × 40 mm were prepared and were sterilized with a UV-C lamp before experiment. The volume of 0.6 ml microbial suspension was applied using a pipette onto the model and historic leather samples, and then the samples were dried in a laminar flow cabinet until a constant mass was obtained.

### 2.3 Application of ethanol in the mist form

The method of applying the ethanol in the mist form to the surfaces of historical objects was established, optimized and described before (Wawrzyk et al., 2023). The aforementioned publication contains information on the process of optimizing the application of ethanol mist, but the research was conducted on textile material. The cotton used in those studies is a cellulose material that has different properties and reacts differently with biocides than the collagen materials included in this publication. Briefly, the Paasche VL 0819 and VE 0707 dual-action airbrush was selected, which allowed the most even spraying of 90% ethanol in the form of a mist, assessed visually as the degree of surface wetting, at a working pressure of 0.2 MPa and using a PA HEAD VLH-5 nozzle with a 1.05 mm diameter tip. Ethanol was applied to the material in the form of a mist from a distance of 16 cm from the sample for 12 seconds on a surface of 16 cm^2^. As part of the optimization of disinfection parameters after applying ethanol mist, the samples were additionally stored in PE (Polyethylene) foil at 21°C ± 1°C for 22 ± 1 h to extend the effect of ethanol vapors. The distance between airbrush and sample was optimized to obtain the most uniform application possible with little wetting and the smallest possible losses resulting from ethanol dispersion.

### 2.4 Evaluation of the biocidal effectiveness of ethanol in the form of mist

After application of ethanol, the samples were allowed to dry for approximately 5 h in a vertical laminar airflow cabinet. Inoculated skin samples without ethanol mist application were used as a control. The microorganisms were then washed out by shaking for 5 min at 250 rpm in 100 ml of peptone water with the addition of 2% Tween. Ten-fold dilutions were made and inoculated onto media. Fungi were incubated on SDA (Sabouraud Dextrose Agar) medium for 5 days at 25°C, and bacteria on PCA medium for 48 h at 30°C. Then, the number of microorganisms was determined using the formula:


(1) $$\begin{array}{l} M=\frac{\Sigma \;C}{v(n_{1}+0.1n_{2})d}\cdot 100 \end{array}$$

Where:

M - number of bacteria/fungi on the sample [cfu/sample];

C- sum of colonies on all plates from the counted dilution;

v- volume of inoculation applied to each plate in ml;

n_1_- number of plates from the counted dilution;

n_2_- number of plates from the second counted dilution;

d- dilution index corresponding to the counted dilution.

If no colonies were detected on two plates, the result was given as <100 or on a logarithmic scale <2. This means that no microorganisms were present or were below the detection limit.

From the obtained number of microorganisms, the percentage and logarithmic reduction were calculated:

The percentage reduction was calculated using the formula:


(2) $$\begin{array}{l} Reducion,\%=\left(W-T\right)/W\cdot 100 \end{array}$$

The logarithmic reduction was calculated using the formula:


(3) $$\begin{array}{l} Reduction\;log10=logT-logW \end{array}$$

Where:

W - number of bacteria on the model sample without alcohol application examined at the same time as the samples with ethanol application.

T - number of bacteria on the model or historical sample after ethanol exposure.

### 2.5 Analysis of the chemical composition of the surface after decontamination

#### 2.5.1 Scanning electron microscopy

The examination using an electron microscope enabled imaging of the surface topography of the analyzed leather samples before and after the disinfection process. The device used in this study was a scanning electron microscope SEM: FEI Quanta 3D FEG with a vacuum sputtering device Polaron SC7640 (Quorum Technologies, Great Britain).

#### 2.5.2 Fourier transform infrared spectroscopy

In order to investigate whether ethanol caused adverse changes on the surface of the disinfected leather, Fourier-transform infrared spectroscopy (FTIR) was applied, using a Nicolet 8700 FTIR spectrometer (Thermo Scientific, USA) with a Smart Orbit™ diamond ATR (Attenuated Total Reflectance) module and MCT (Mercury Cadmium Telluride) detector cooled with liquid nitrogen. Before and after the application of ethanol in the form of mist, spectra were collected from a 2–3 μm thick layer in the range of 4,000–650 cm^-1^ with a resolution of 4 cm^-1^. The ATR spectra were subjected to ATR correction, scaled normalization, and baseline. Spectra equivalent to transmission spectra were obtained. The OMNIC 3.2 software (Thermo Scientific, USA) was used for the tests.

#### 2.5.3 X-Ray photoelectron spectroscopy

The determination of the chemical composition of the surface layer of the samples before and after the decontamination process with 90% ethanol solution was carried out using the XPS technique. For this purpose, a multi-chamber UHV (Ultra-High Vacuum) analytical system (Prevac, Poland) was used for the measurement. The photoelectrons were excited by X-ray radiation with the characteristic line Al Kα and energy of 1486.7eV, generated by the VG Scienta SAX 100 lamp with an aluminum anode together with the VG Scienta XM 780 monochromator. The operating parameters of the X-ray lamp used: U = 12 kV, Ie = 30 mA. The photoelectrons were recorded using the Scienta R4000 hemispherical analyzer. The pressure in the analysis chamber during the measurements was maintained at a level lower than 1.0E-8mbar. Basic parameters of the analyzer for the survey spectrum: operating mode: sweep, transition energy: 200eV, measured range of photoelectron binding energy: 0–1350eV, measurement step: 0.5eV, collection time in a single step: 200ms. In order to compensate for the electric charge generated during the measurement, the samples were bombarded with a beam of low-energy electrons. The recorded spectra were processed using the Casa XPS Version 2.3.16 PR16 program. All spectra were calibrated by determining the position of the C1s carbon line for the energy of 284.5eV.

## 3 Results

### 3.1 The number of microorganisms on leather objects

The total number of bacteria on the historical children's shoes from A-BSM collections was 1.3 × 10^3^ CFU/100cm^2^. Fungi contaminated these surfaces to similar extent as bacteria −1.26 × 10^3^ CFU/100cm^2^.

### 3.2 Biocidal efficacy of ethanol in the form of mist

For each of the tested strains, a reduction of microbial counts of more than 99.5% was demonstrated. The highest reduction of 4.1 log was observed for the *Staphylococcus aureus* strain. The lowest reduction of 2.31 log was observed for the *Alternaria alternata* strain. The results are presented in the Table 2.

**Table 2:** Evaluation of the biocidal effectiveness of ethanol against selected strains for model and historic leather.

**Strain**	**Sample type**	**Mass of ethanol applied [g]**	**Cell count [cfu/sample]**	**log [cfu/ sample]**	**Reduction level**	
					**log**	**%**
*Alternaria alternata (A-BSM cryobank)*	Model	0	2.05 × 10^4^	4.31	-	-
	Model	0.20	1.0 × 10^2^	2.0	2.31	99.51
	Historical	0.16	1.0 × 10^2^	2.0	2.31	99.51
*Aspergillus flavus (ATTC 9643)*	Model	0	1.9 × 10^5^	5.26	-	-
	Model	0.18	9.5 × 10^1^	2.0	3.26	99.95
	Historical	0.21	9.5 × 10^1^	2.0	2.36	99.95
*Aspergillus niger (A-BSM cryobank)*	Model	0	6.0 × 10^4^	4.78	-	-
	Model	0.11	2.04 × 10^2^	2.0	2.48	99.66
	Historical	0.22	1.02 × 10^2^	2.0	2.78	99.83
*Pantoea agglomerans (A-BSM cryobank)*	Model	0	6.77 × 10^4^	4.83	-	-
	Model	0.21	1.02 × 10^2^	2.00	2.83	99.85
	Historical	0.16	1.02 × 10^2^	2.00	2.83	99.85
*Penicillium chrysogenum (A-BSM cryobank)*	Model	0	1.04 × 10^5^	5.02	-	-
	Model	0.19	1.04 × 10^2^	2.0	2.98	99.90
	Historical	0.13	1.04 × 10^2^	2.0	2.98	99.90
*Staphylococcus aureus (ATTC 6538)*	Model	0	1.25 × 10^6^	6,1	-	-
	Model	0.13	1.25 × 10^2^	2.00	4.10	99.99
	Historical	0.12	1.25 × 10^2^	2.00	4.10	99.99

### 3.3 Analysis of surface topography and morphology

#### 3.3.1 Scanning electron microscopy

**Figure 3:**
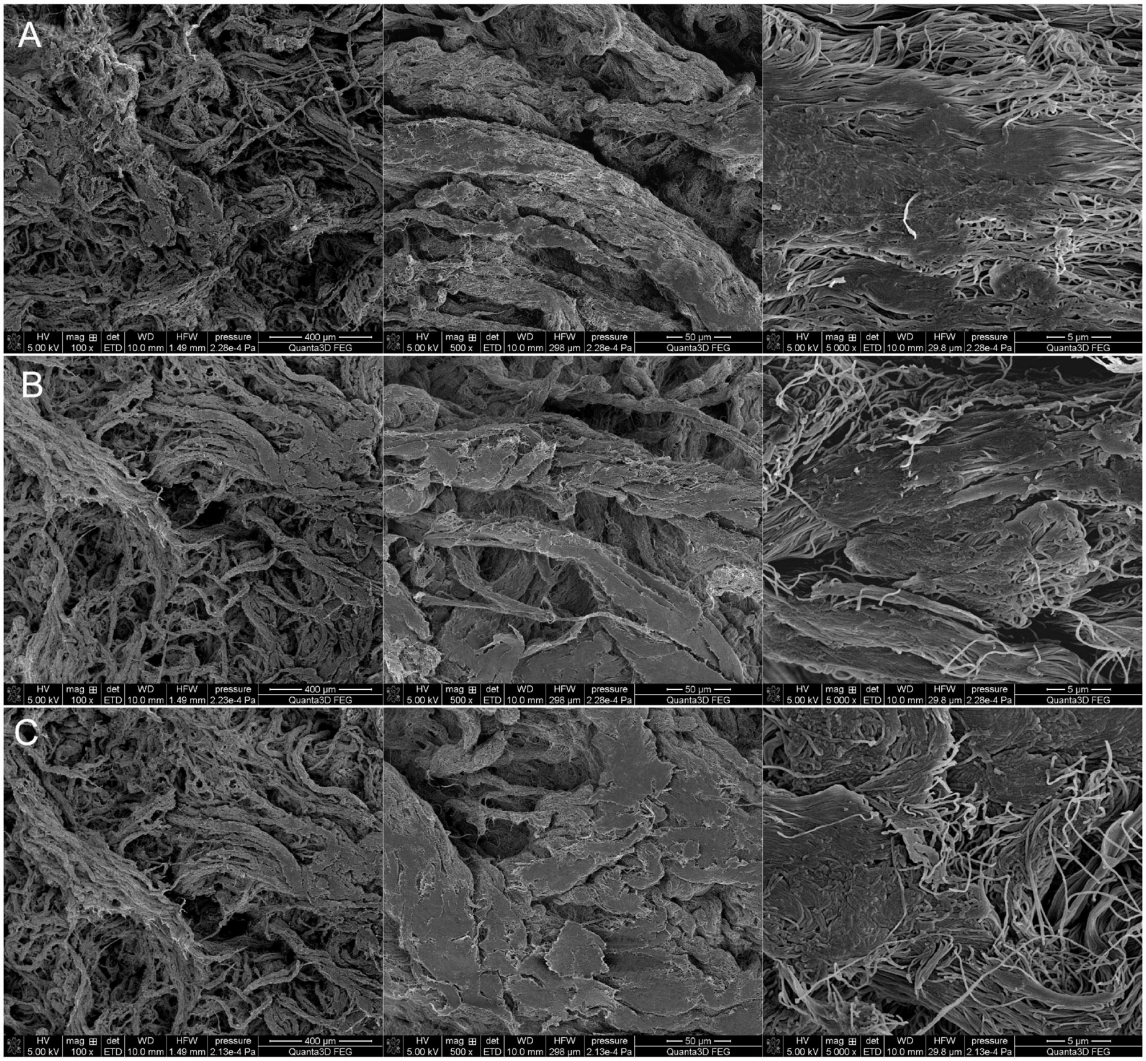
Scanning Electron Microscopy (SEM) microscopic images of model leather at magnifications of 100, 500, and 5,000x: **(A)** model leather—control, **(B)** model leather—after application of 90% ethanol in the form of mist and direct drying, **(C)** model leather—after application of 90% ethanol in the form of mist and drying after 22 h of storage in ethanol vapors.

**Figure 4:**
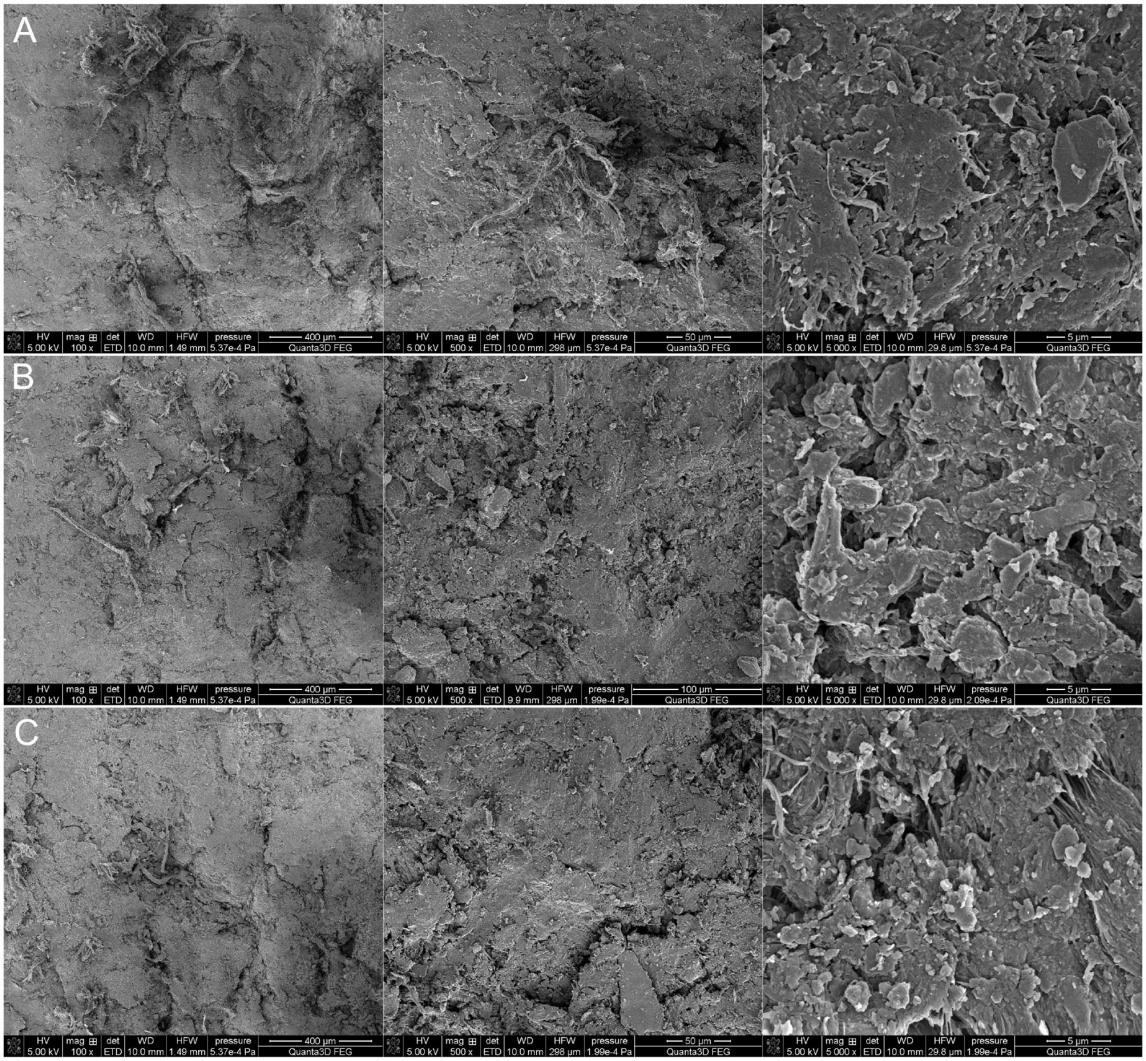
Scanning Electron Microscopy (SEM) microscopic images of historical leather at 100, 500, and 5,000x magnifications: **(A)** historical leather—control, **(B)** historical leather—after application of 90% ethanol in the form of mist and direct drying, **(C)** historical leather—after application of 90% ethanol in the form of mist and drying after 22 h of storage in ethanol vapors.

In order to visualize the changes in surface topography and morphology under the influence of 90% ethanol in the form of mist applied to model and historical leather, dried immediately after application and after 22 h of storage in a foil package, examinations were performed under a SEM microscope. The model leather I and historic leather I samples were control samples that were not subjected to the decontamination process. The model leather II and historic leather II samples were prepared by applying a 90% ethanol solution to the surface, and the measurement was performed immediately after the biocidal agent had been applied and dried. For the model leather III and historic leather III samples, the measurement was performed 22 h after disinfection with ethanol in the form of a mist after 22 h of storage in ethanol vapors. Microscopic examinations conducted using scanning electron microscopy on both model (Figure 3) and historical (Figure 4) leather samples did not reveal any significant differences in the structure of the tested material in the analyzed samples. Moreover, the disinfection method used did not have a significant effect on the surfaces of the tested materials. No noticeable changes in the morphology of the observed surfaces were found. It can therefore be concluded that the disinfection technique used did not adversely affect the physicochemical properties of the collagen fibers contained in the disinfected samples.

#### 3.3.2 Fourier transform infrared spectroscopy

The model leather I and historic leather I samples were control samples that were not subjected to the decontamination process. The model leather II and historic leather II samples were prepared by applying a 90% ethanol solution to the surface, and the measurement was performed immediately after the biocidal agent had been applied and dried. For the model leather III and historic leather III samples, the measurement was performed 22 hours after disinfection with ethanol in the form of a mist after 22 hours of storage in ethanol vapors. For all samples, the measurement was performed on the top and bottom side of the sample.

**Figure 5:**
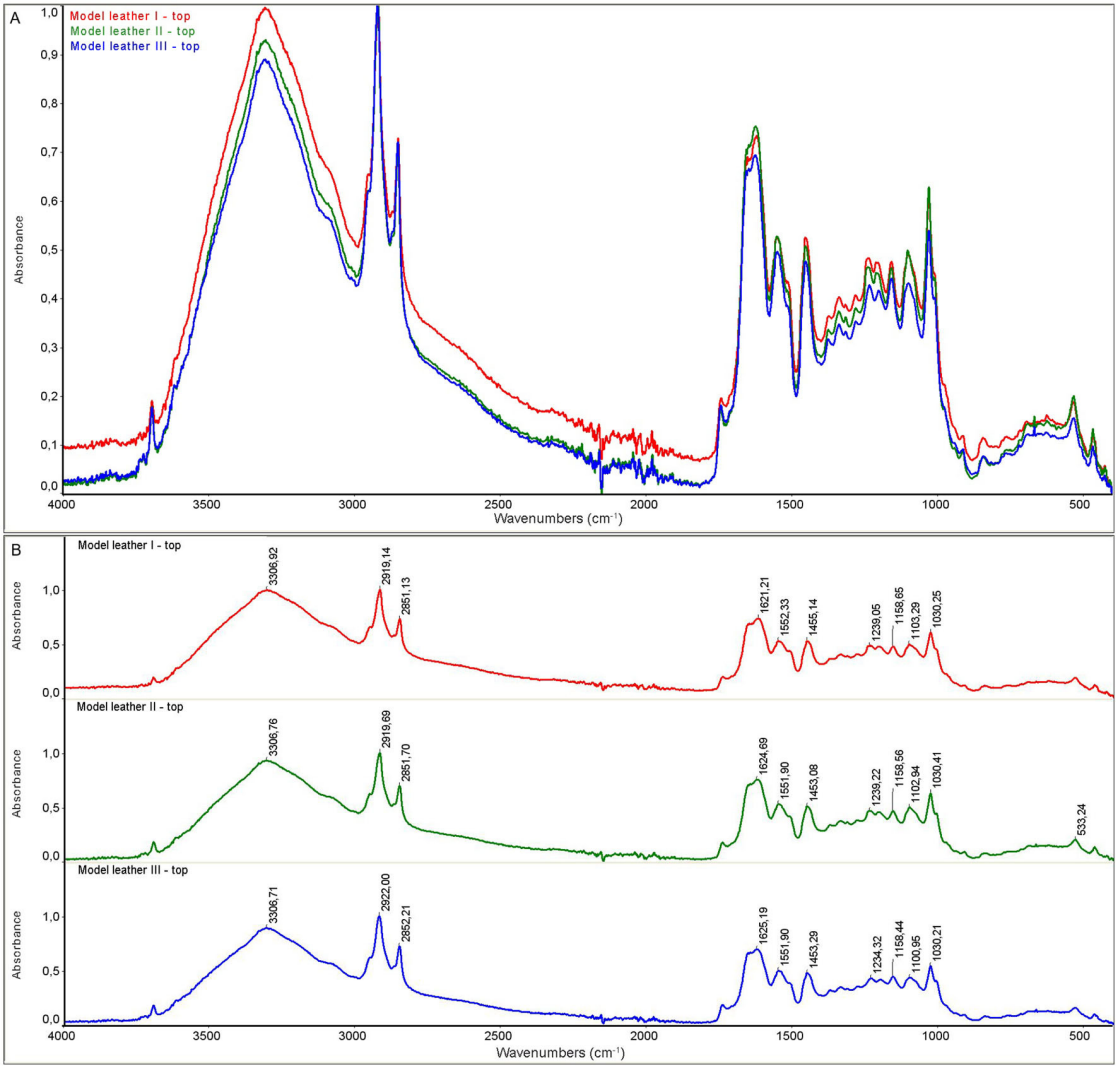
ATR spectra of the model leather samples, upper side: **(A)** superimposed, **(B)** with characteristic peaks marked; model leather I (red)—not subjected to the decontamination process; model leather II (green)—after applying ethanol and dried; model leather III (blue)—after 22 h of storage in ethanol vapors.

**Figure 6:**
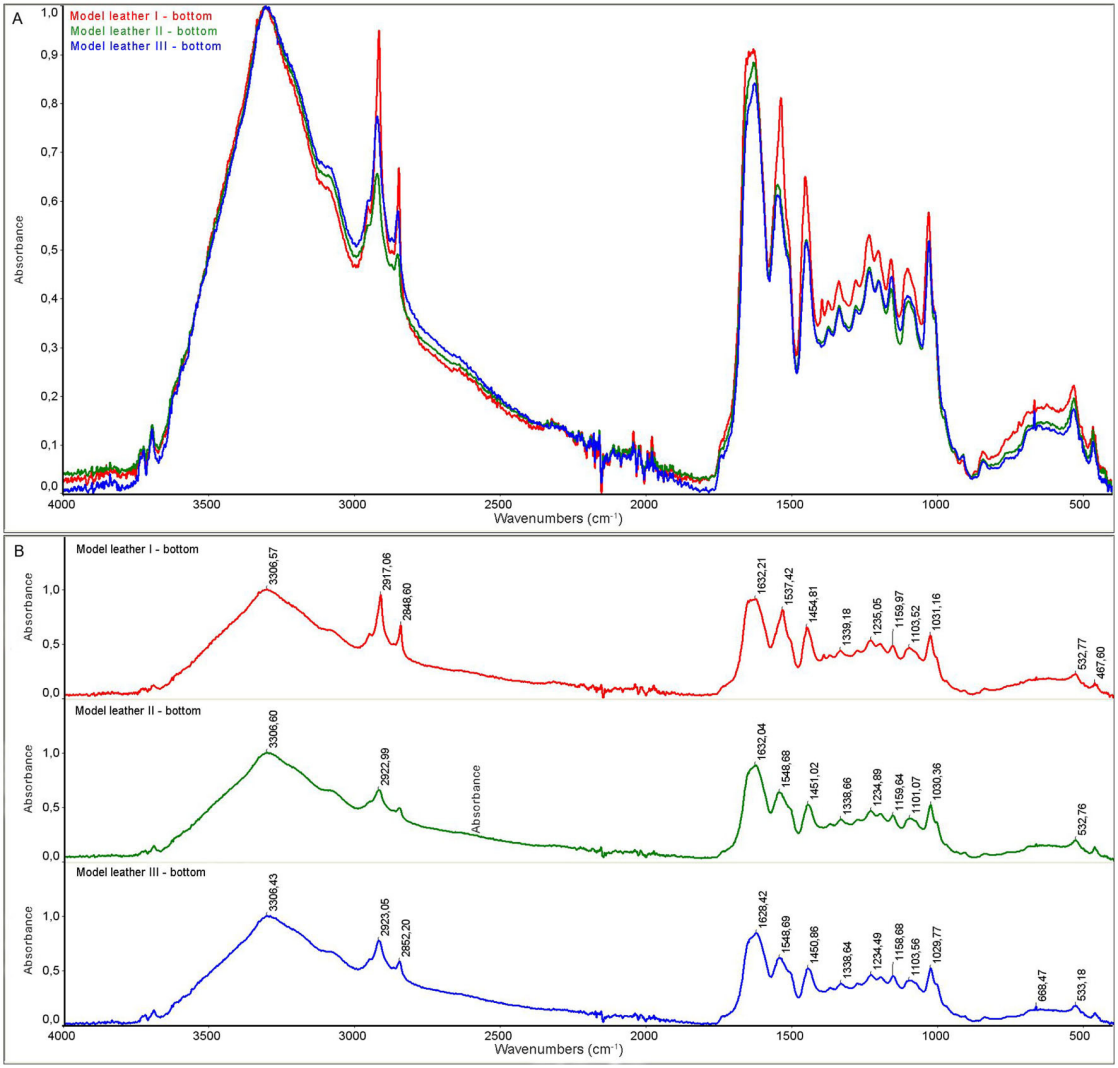
ATR spectra of the model leather samples, back side: **(A)** superimposed, **(B)** with characteristic peaks marked; model leather I (red)—not subjected to the decontamination process; model leather II (green)—after applying ethanol and dried; model leather III (blue)—after 22 h of storage in ethanol vapors.

**Figure 7:**
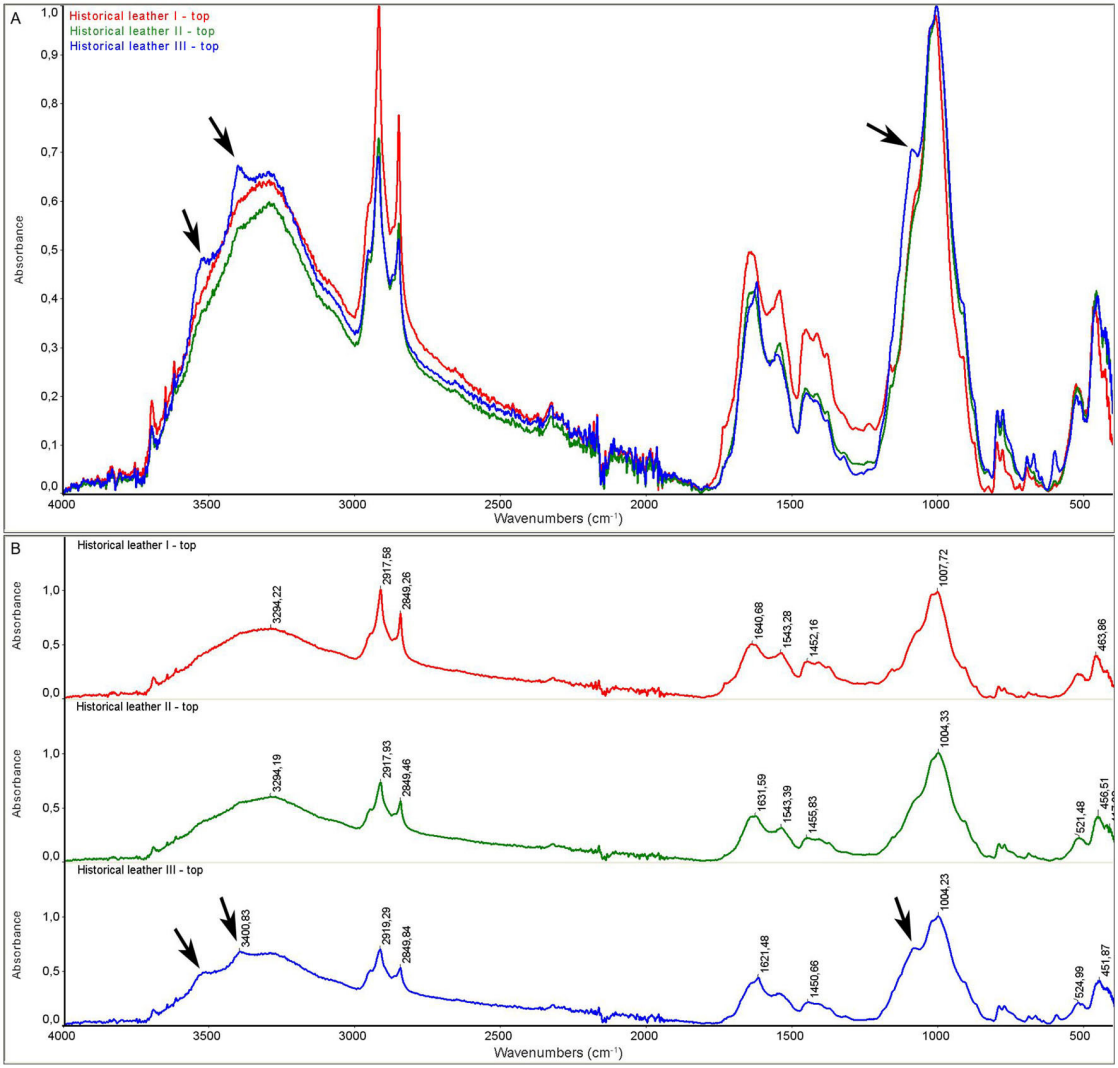
ATR spectra of historical leather samples, top side: **(A)** superimposed, **(B)** with characteristic peaks and new ones marked with arrows; historical leather I (red)—not subjected to the decontamination process; historical leather II (green)—after applying ethanol and dried; historical leather III (blue)—after 22 h of storage in ethanol vapours.

**Figure 8:**
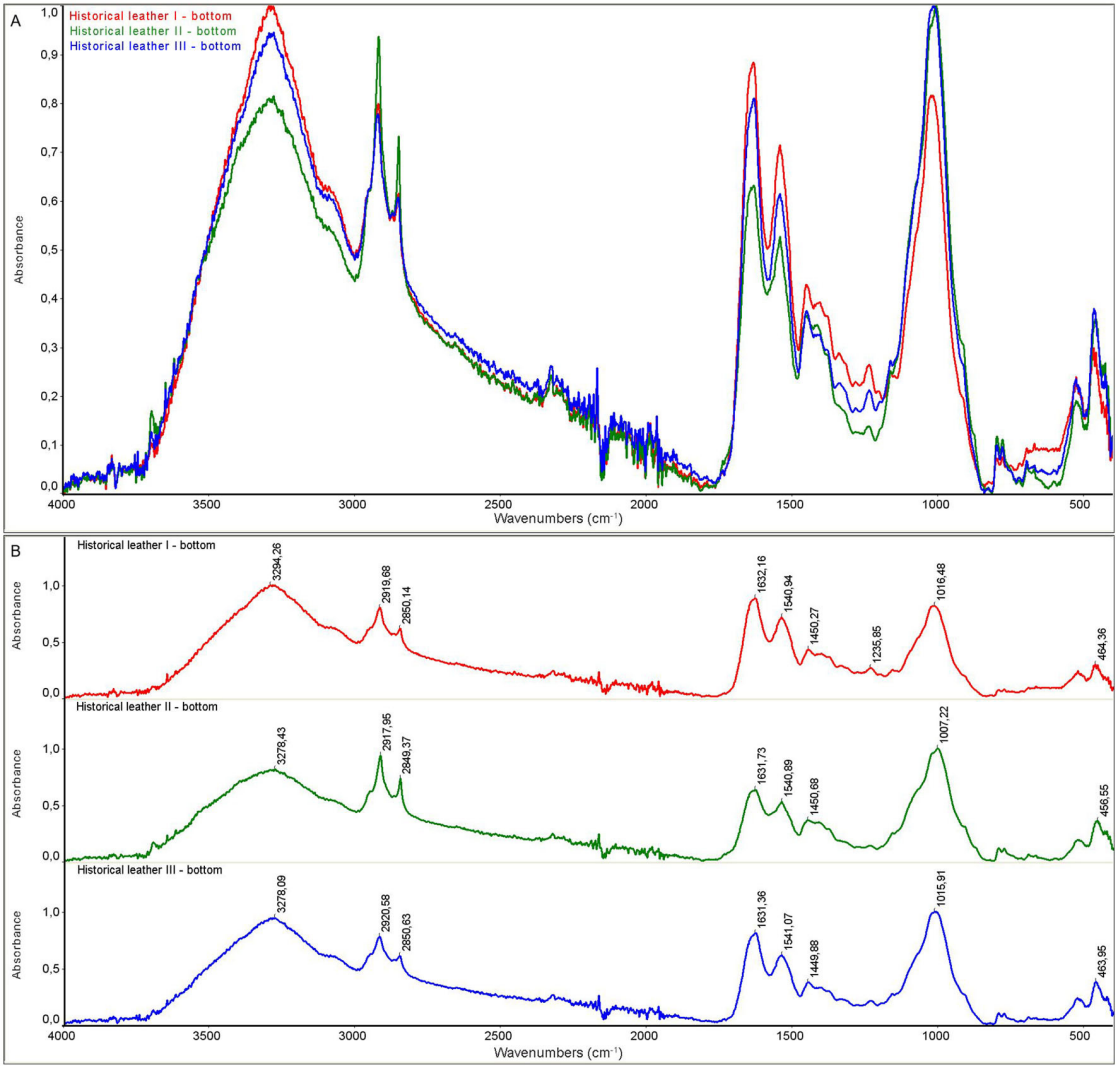
ATR spectra of historical leather samples, back side: **(A)** superimposed, **(B)** with characteristic peaks marked; historical leather I (red)—not subjected to the decontamination process; historical leather II (green)—after applying ethanol and dried; historical leather III (blue)—after 22 h of storage in ethanol vapours.

As a result of the measurements, the spectra of the model material samples and the spectra of the historic material were obtained. The spectra for the model material samples are shown in Figures 5, 6. FTIR-ATR studies of model leather samples I, II, III showed slight differences in the intensity of the IR spectral bands. No changes in the band positions were observed due to the use of ethanol. Figures 7, 8 show the spectra obtained for the historic leather samples. For the historical leather top sample after the use of ethanol exposed to long-term exposure to ethanol vapors, a slight change in the band intensity was observed in relation to the reference sample—new signals were observed in the area of 3,500 cm^–1^, 3,400 cm^-1^, 1,200–1,100 cm^–1^ (Figure 7). The peaks of historical leather I, historical leather II and historical leather III, located at the same wavenumber value, overlap, which indicates no significal differences between the samples treated with ethanol and not treated (Figures 7, 8).

#### 3.3.3 Analysis of the chemical composition of the surfaces

**Figure 9:**
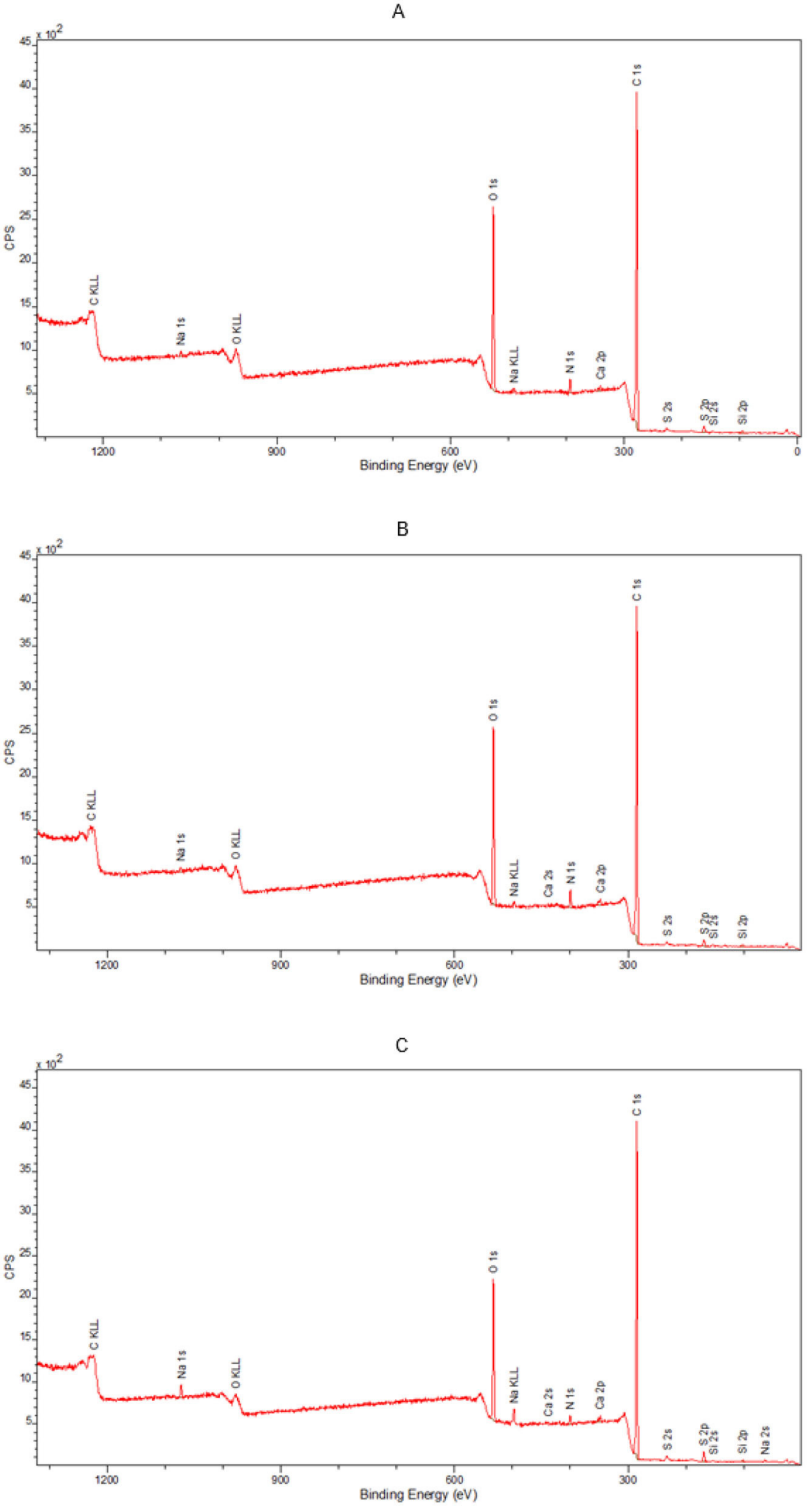
Broadband spectrum obtained by XPS spectroscopy for samples: **(A)** model leather I; **(B)** model leather II; **(C)** model leather III, used to calculate the elemental composition of the surface of the tested material.

**Figure 10:**
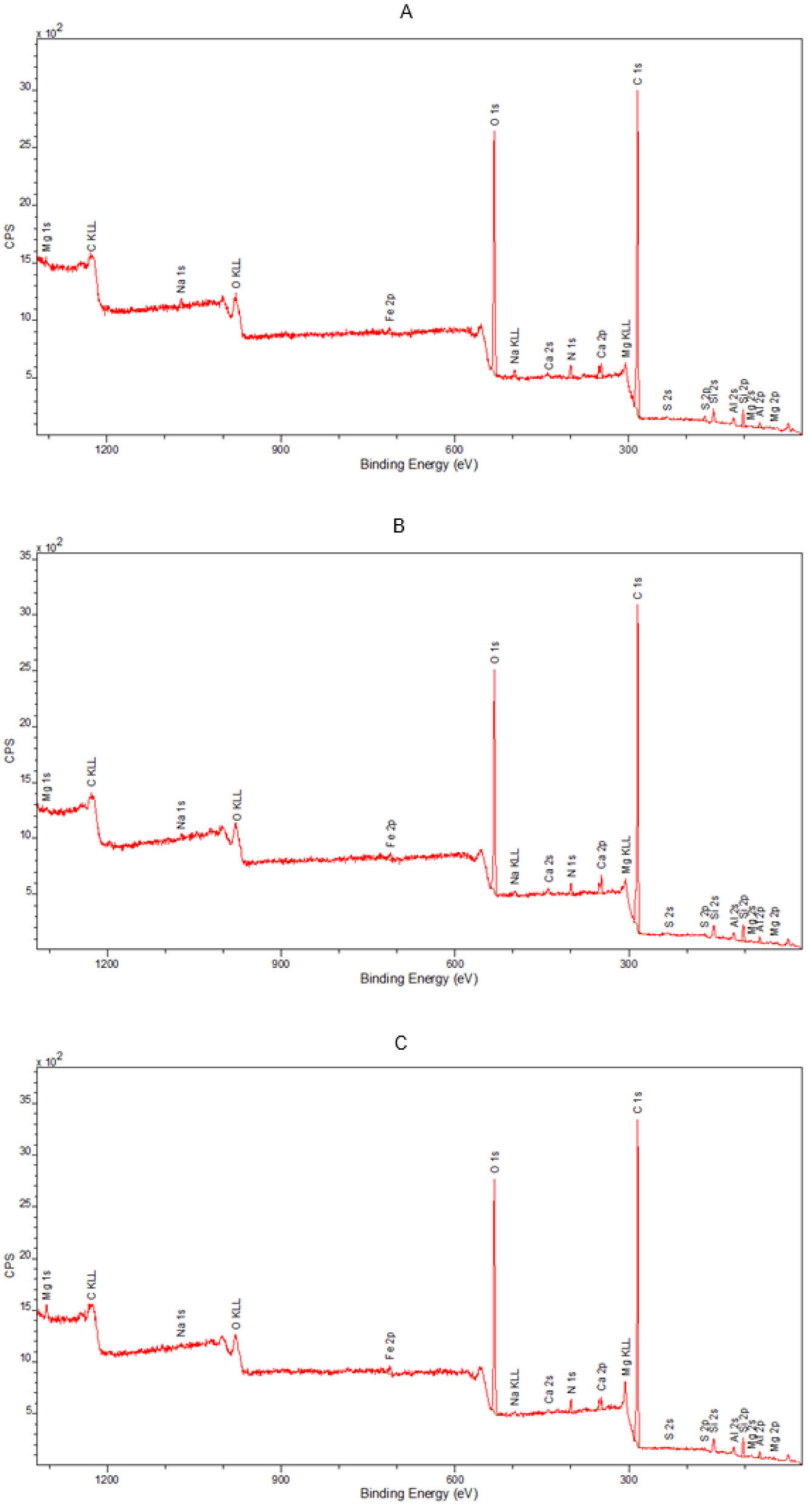
Broadband spectrum obtained by XPS spectroscopy for the samples: **(A)** historic leather I; **(B)** historic leather II; **(C)** historic leather III, used to calculate the elemental composition of the surface of the tested material.

**Figure 11:**
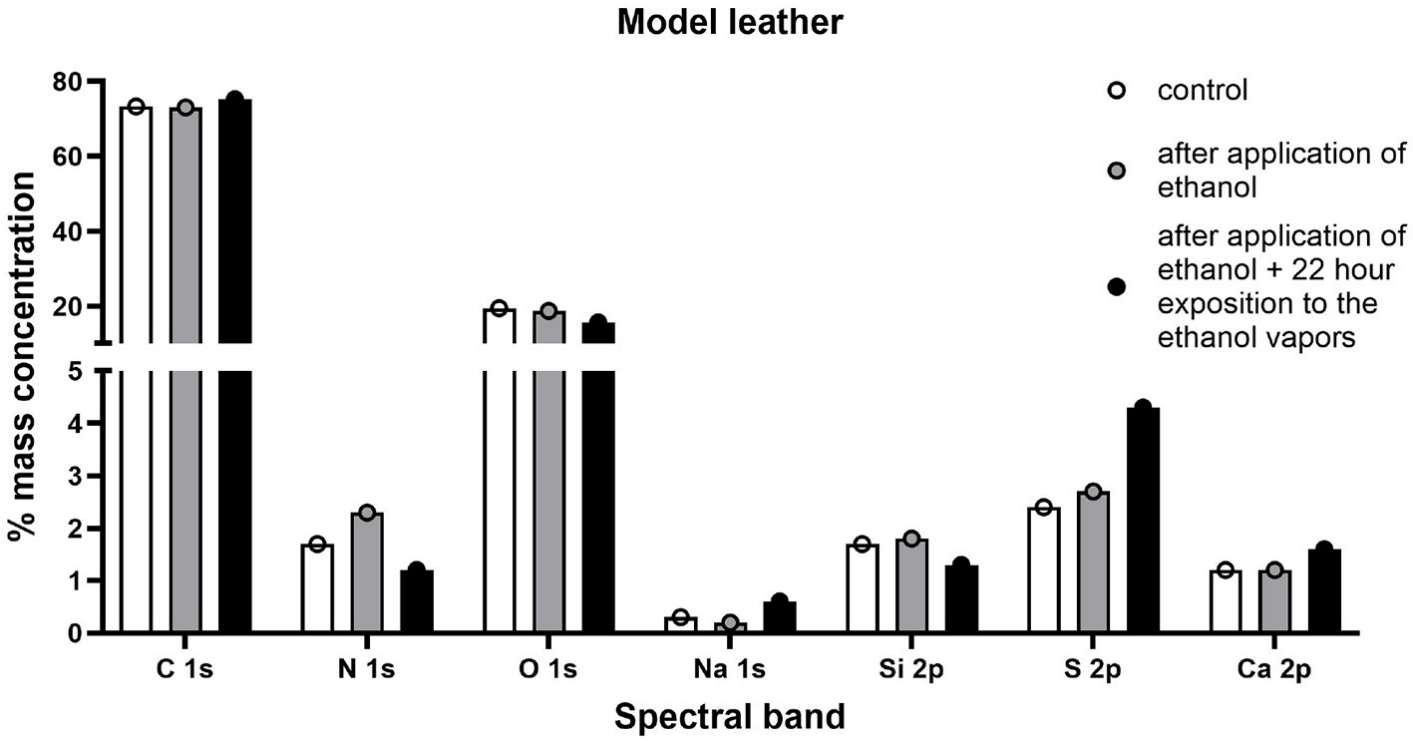
Percentage distribution of individual elements included in the outer layer of the model leather samples subjected to the test.

In order to fully illustrate the effect of disinfection with a 90% ethanol solution applied in the form of a mist on the leather surfaces, an additional study was performed using the XPS technique to determine the elemental composition of the outer layers of the tested samples. Analysis of the spectral lines visible in the spectra enabled the detection of elements contained in the tested samples. The outer surface of the model leather contained the following elements: carbon, nitrogen, oxygen, sodium, silicon, sulfur, calcium, and phosphorus (Figure 9). The samples of historic leather contained carbon, nitrogen, oxygen, sodium, aluminum, magnesium, silicon, sulfur, calcium, and iron on their surface (Figure 10). In model leather samples, the vast majority of the elemental composition expressed in mass percentages is carbon and oxygen, which is due to the structure of the leather, mostly composed of collagen (Figure 11). XPS studies performed in a narrow range of binding energies showed the presence of the following chemical bonds characteristic of carbon: C-C, C-O-C, C=O, O-C=O. Calcium present in the samples occurs in the form of carbonates, while silicon in the form of silicon(IV) oxide. No significant differences were found between not treated and decontaminated model leather samples (Figure 9).

**Figure 12:**
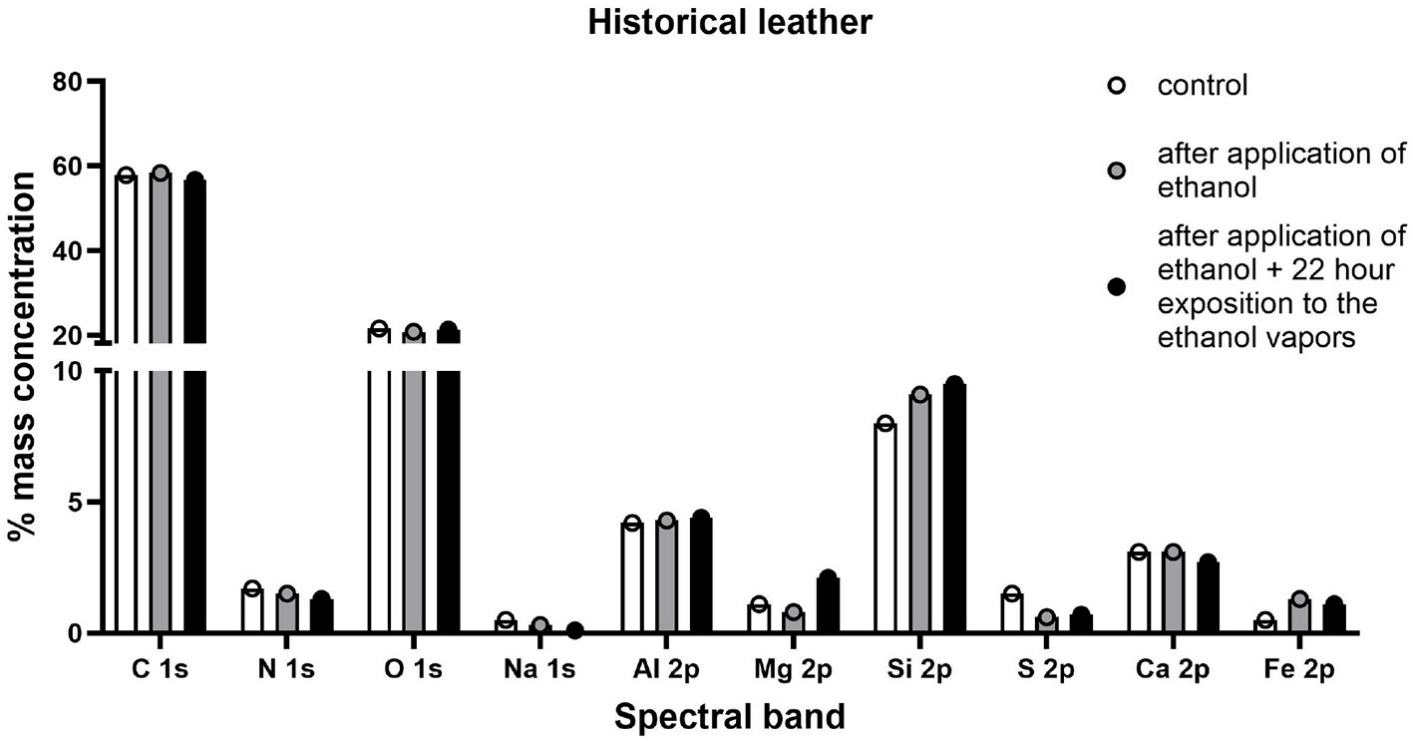
Percentage distribution of individual elements included in the outer layer of the tested historical leather samples.

In the case of historic samples, among elements found in model leather, aluminum, iron and sulfur were also determined, which most likely constitute a secondary layer of the tested historic surface. The average analysis depth for an XPS measurement is about 5 nm, so it mainly determines changes in the secondary layer of the object. Analysis of samples of historic leather materials also did not show any significant changes in the composition of elements after disinfection with a 90% ethanol solution in the form of mist (Figure 12).

XPS studies performed in a narrow range of binding energies showed the presence of the following chemical bonds characteristic of carbon: C-C, C-O-C, C=O, O-C=O, C-C, C-O-C, C=O, O-C=O, C-C/C-HC-C/-C-N-, C-OH, C-O-C, C=O, COOR, C-C/C-H, C-C/ -C-S-/-C-N-, C-OH, C-O-C, C=O, and COOR. Calcium present on the surface of the tested samples occurs in the form of carbonates, while silicon in the form of SiO_2_.

## 4 Discussion

Leather is a common building material for historical objects found in A-BSM, such as shoes or suitcases, which are often the only remnant of their owners, victims of the concentration camp, and therefore their historical value is priceless.

Microorganisms that may be harmful to human health and have a destructive effect on the surfaces of historical objects are identified on objects found in A-BSM. The presence of bacteria and fungi, native to the museum environment, on the surfaces of historical objects may pose a threat to the health of people exposed to professional contact with such items (Skóra et al., 2015). Biodeterioration of leather objects involves the decomposition of tannins, fats and proteins and may result in the appearance of discolorations, stains, indentations and a weakening of the strength (Rybitwa et al., 2020). In the current study, we detected an average of 1.26 × 10^3^ cfu/100cm^2^ of bacteria and 1.30 × 10^3^ cfu/100cm^2^ of fungi on the leather surfaces of historical children's shoes. In addition, in 2020 we published data on microbiological contamination of leather objects. On the surfaces of leather shoes, the average number of bacteria ranged between 1.96 × 10^6^ and 7.61 × 10^6^ cfu/100cm^2^, while fungi colonized these objects in an average number of 2.33 × 10^4^–2.56 × 10^4^ cfu/100cm^2^. Thirty species of bacteria and 24 species of fungi were identified, including microorganisms that could adversely affect the state of preservation of the objects (e.g., *P. agglomerans, A. pullulans*) and the health of workers carrying out conservation work (e.g., *B. cereus, S. epidermidis*) (Rybitwa et al., 2020). For comparison, public utility areas are characterized by presence of microorganisms in average number ranging from 10^1^ to 10^4^ cfu/m^2^ (Wawrzyk et al., 2020a). Uneven distribution of microorganisms on historical leather objects may result from various factors such as the chemical composition of the leather, its degree of preservation, biocides used in the past and environmental conditions. Well-preserved leather (e.g., impregnated with fats, waxes) is less susceptible to colonization, because microorganisms have a harder time breaking down hydrophobic protective substances. Damaged, dried out or cracked leather creates microenvironments that are conducive to the deposition of dust and moisture, which facilitates the development of microorganisms.

Based on the results of previous studies (Rybitwa et al., 2020), to analyze the biocidal effectiveness of ethanol in the form of mist, we selected microorganisms occurring on the surfaces of leather objects in A-BSM, potentially harmful to objects and human health, such as *Aspergillus flavus, Aspergillus niger, Pantoea agglomerans*, and *Penicilium chrysogenum*. Bacteria of the *Staphylococcus* genus were also previously identified on leather objects (Rybitwa et al., 2020), so for our research we used a health-threatening representative of this group, namely *Staphylococcus aureus*. In addition, the mold fungus *Alternaria alternata*, often found on historical objects in A-BSM (Koziróg et al., 2014; Rajkowska et al., 2014; Wawrzyk et al., 2020b, 2018), which produces acids contributing to the progress of biodeterioration (Di Carlo et al., 2016) and may also cause allergies in humans, was selected for our study.

Biodegradation is a major problem in the protection of cultural heritage, including historical leather objects, and traditional biocides often have limited effectiveness due to microbial resistance (Vadrucci et al., 2020). A commonly used method of disinfecting microbiological contamination of abiotic surfaces is spraying with 70% ethanol. It should be noted that this method is rarely used in museums, because contact of aqueous solutions with historical surfaces may adversely affect their surface, and ultimately negatively affect its properties. As a result of wetting, the leather surface may shrink, stiffen, which leads to increased potential fragility or a noticeable change in morphology. In earlier attempts described in the literature, in order to use ethanol on historical leather, tests were performed using 70%–80% alcohol. However, promising results were obtained only as a result of applying a compress for several hours and soaking the leather sample for 10 min in an aqueous alcohol solution. Satisfactory effectiveness was achieved, although the author does not recommend this method for use on cultural heritage objects (Scheerer, 2012). Therefore, it is important to constantly search for alternative techniques that will eliminate microorganisms and, at the same time, be safe for objects containing collagen materials, enabling safe work of conservators (Dignard and Mason, 2020).

The primary objective of the study was to test the biocidal efficacy of ethanol in the form of mist applied to historical leather materials, as well as its effect on modern leather materials. In previous studies, we demonstrated almost 100% effectiveness of disinfection of textile, cotton historical objects using 90% ethanol in the form of mist, without damaging their surfaces (Wawrzyk et al., 2023, 2024). We also used 90% ethanol in the form of mist to reduce autochthonous microflora occurring on paint layers in historical buildings located in the A-BSM area. Thanks to this approach, the quantitative reduction of bacteria was achieved in the range of 70.4%–>99%, and fungi in the range of 78.2%–>99% (Wawrzyk et al., 2025). In order to optimize the disinfection process and achieve a greater reduction in the number of microorganisms, the method used in the conservation of historic objects for paper disinfection was used: the time of ethanol vapor exposure was extended to 22 h (Karbowska-Berent et al., 2018). Our studies showed >99% reduction in the number of all tested fungi (*Aspergillus flavus, Aspergillus niger, Penicilium chrysogenum*, and *Alternaria alternata*) and bacteria (*Pantoea agglomerans, Staphylococcus aureus*) present on both model and historic leather after disinfection with ethanol in the form of mist.

However, in museums, the implementation of innovative disinfection techniques requires a thorough examination of the potential consequences associated with such a process. In order to assess the possible harmfulness of the disinfection method using 90% ethanol in the form of mist on the surfaces of historical objects, a number of analyses were performed. The use of SEM microscopy allows for the detection of surface damage and collagen structure cracks, which could lead to mechanical weakening of the object, through the loss of specific material properties and constitute a place for initiation of the adhesion of microorganism cells, leading to further biodeterioration. Scanning Electron Microscopy (SEM) is a proven method for imaging cracks on surfaces in other areas of science (Wawrzyk et al., 2021). The images obtained during SEM observations did not show significant changes for samples in this regard after using ethanol as a disinfection method.

Collagen-based materials are very sensitive to degradation and are mainly subject to hydrolysis and oxidation. Namely, acid hydrolysis and photo-oxidation are the most common causes of degradation of leather products. Several studies on parchment have described typical features of hydrolysis, oxidation and gelatinization observed using ATR-FTIR spectroscopy, which is widely used in collagen degradation studies. ATR-FTIR spectroscopy can detect changes in the amide I and amide II bands, with an increase in the distance between wavenumbers and the intensity ratio indicating oxidation, while changes in the AI/AII ratio can indicate the type of hydrolysis (Vyskočilová et al., 2019). FTIR measurements of examined samples did not reveal any negative effect of disinfection with 90% ethanol in the form of mist on the structure of disinfected leather samples, both model and historical ones. Obtained results indicate that the ethyl alcohol used in the study evaporated to a large extent from the tested surfaces. Leather products consist of collagen fibers, which do not react significantly with ethanol.

Complementary to FTIR measurements, XPS tests confirm the lack of influence of disinfection of leather samples with a 90% ethanol solution in the form of mist on the chemical structure of the outer surface layer. The slight changes in the percentage content of individual elements could have been caused by the presence of ethanol on the surface of the samples, which did not evaporate completely.

The obtained results allow to demonstrate the lack of contraindications to the use of this technique for disinfection of historical leather materials. Disinfection with ethanol applied in the form of mist after optimizing the concentration, exposure time and selection of the appropriate application technique can be effectively used to eliminate microbiological contamination of historical objects made of leather. What is important, like every method dedicated to use on historical material, it requires validation for specific objects.

The ethanol mist method does not require large financial outlays, complicated equipment, or special precautions compared to other methods used to protect cultural assets. Minimizing mechanical damage reduces the risk of physical damage to the leather surface. The mist form can allow for even distribution of the substance, reaching hard-to-reach places. It is a quick and easy-to-use method in museum, so it can be practically implemented in current conservation tasks. Ethanol evaporates quickly, which can reduce the risk of long-term exposure to moisture on the material, and therefore can be used to disinfect materials that should not be exposed to aqueous solutions.

Ethanol effectively eliminates many types of microorganisms, although as a disinfectant has limited effectiveness compared to other waterless disinfection techniques because it requires the presence of water to effectively denature proteins and its rapid evaporation may leave microbial spores intact, whereas methods such as gas disinfection (e.g., ethylene oxide) or UV radiation are more effective against a broad spectrum of microorganisms, including spores. However, ethanol is less toxic and less harmful to humans compared to methods using ethylene oxide or ionization, which can be effective in eliminating pathogens on surfaces, but have limited use in decontamination of heritage objects.

To conclude, we proved that disinfection of historical leather objects with the 90% ethanol in the mist form is biocidally effective, for tested bacteria and fungi. Moreover, we were first to demonstrate that this technique does not damage the leather object's surface structure, as examined by SEM, FTIR, and XPS analyses. 90% ethanol evaporates quickly, so there is no threat of soaking of the surface. This disinfection method is also cheap, easy to use, convenient and highly available. What is important, it requires validation before use on different historic materials.

## Data Availability

The raw data supporting the conclusions of this article will be made available by the authors, without undue reservation.
